# Some Applications of Vibrational Spectroscopy for the Analysis of Polymers and Polymer Composites

**DOI:** 10.3390/polym11071159

**Published:** 2019-07-08

**Authors:** Liliane Bokobza

**Affiliations:** 196 Boulevard Bineau, 92200 Neuilly-Sur-Seine, France; liliane.bokobza@wanadoo.fr; Tel.: +33-1-4637-2427

**Keywords:** vibrational spectroscopy, infrared, near-infrared, Raman, polymers, polymer processes, orientation, nanocomposites

## Abstract

Vibrational spectroscopies, including infrared and Raman techniques, are important tools for the characterization of chemical composition, molecular structures, and chain orientation under mechanical deformation of polymeric materials. The development of fiber-optic-based spectrometers has broadened the use of vibrational spectroscopy for process monitoring in various fields including polymerization, curing, and manufacturing processes. Combined with chemometrics, near-infrared (NIR) spectroscopy is now recognized as one of the most important techniques for polymer analyses. Infrared and Raman studies also offer invaluable means for the analysis of inorganic particles used as reinforcing fillers for polymers. The characterization of surface species and the nature of interfacial bonding between the organic and inorganic phases are important issues for the understanding of composite properties. Infrared spectroscopy is particularly convenient for the detection and analysis of hydroxyl groups on filler surfaces, and Raman spectroscopy is particularly well suited for the study of carbon-based materials. In both techniques, polymer-filler interactions can be evidenced through frequency shifts or width changes of bands associated with vibrational modes of functional groups of either macromolecular chains or filler particles. Selected examples of application of infrared and Raman spectroscopies illustrate their potential for monitoring polymer processes, measuring polymer orientation, and characterizing polymer composites.

## 1. Introduction

Morphological aspects of polymers such as composition and molecular structure, are well known to determine the physical properties of materials such as glass-transition temperature, crystallinity, mechanical response, or failure phenomenon. In addition to homopolymers, two-phased structures as blends composed of two different polymers or composites containing polymeric and nonpolymeric species, can display improved properties with regard to homopolymers on account of possible synergistic effects arising between the different components. Polymeric materials are usually submitted to mechanical testing including stress–strain behavior at large strains and dynamic mechanical analysis at small strains. The mechanical characterization provides general information on the material and yields important parameters such as Young’s modulus, tensile strength, or molecular motions. It can be complemented by spectroscopic techniques that can afford a deeper understanding of the structures and polymer properties. Moreover, these techniques are non-destructive and provide information at a molecular level. This paper highlights the particular application of vibrational spectroscopy (infrared and Raman) that can allow analysis of homopolymers, copolymers, polymer blends, and polymer composites as well. Control of polymerization and degradation processes, in addition to the characterization of structural properties including tacticity, crystallinity, or orientation, are topics commonly investigated by vibrational spectroscopy. In polymer composites, vibrational spectroscopy can bring information on the filler surface chemistry and the polymer-filler interface, which remain crucial parameters in the reinforcement processes of polymers. This paper is intended to illustrate some applications of infrared and Raman spectroscopies with a special focus on process monitoring, polymer orientation, and characterization of polymer composites.

## 2. Basic Principles of Vibrational Spectroscopy and Sampling Methods

Vibrational spectroscopy, including infrared and Raman spectroscopies, probes the vibrational states of a molecule. Infrared absorption results from a direct resonance interaction between the incident radiation and a particular vibration mode of a molecule, while the Raman effect is an inelastic light scattering process arising upon irradiation of a molecule with a monochromatic light. Infrared activity requires a change in the dipole moment of the molecule while Raman activity requires a change in the polarisability of the molecule during the vibrational mode considered. If the molecule has symmetry elements, application of the group theory allows the determination of the symmetry properties of all vibrational modes and their spectral activity. In particular, if a molecule has a center of symmetry, the vibrational modes symmetric with respect to the inversion operation are Raman active while the antisymmetric ones are infrared active, thus showing the complementarity between the two techniques. Fluorescence arising from impurities has long limited the applicability of the Raman technique. The use of a near infrared excitation source has substantially reduced or removed the fluorescence that interferes with the Raman scattered signal. On the other hand, laser-induced sample heating is a well known problem associated with Raman spectroscopy that can lead to damages in the samples or shifts of some Raman bands as observed in carbon-based materials [1]. This effect can be minimized by decreasing the incident laser power.

The occurrence of almost constant vibrational frequencies associated with the presence of particular chemical functional groups vibrating independently of the rest of the molecule, simplifies the assignment of polymer infrared and Raman spectra. The concept of group frequency is justified by simple mechanical models, which demonstrate that double and triple bond stretching vibrations of high force constant vibrate at their own frequency, and that hydrogen stretching vibrations (X-H) are slightly mechanically coupled to the other vibrators of the molecule. However, a vibrational frequency may be affected by hydrogen bonding, electronic, or resonance effects. For further details on the theoretical aspects of infrared and Raman spectroscopies, the reader should consult References [2,3,4]. 

The samples can be examined in the mid-infrared (mid-IR) range by transmission methods, but in the case of too strong absorption bands, they can be analyzed in the transmission mode too but by near-infrared spectroscopy (NIR) that probes overtones and combination bands much less intensely compared to the fundamental modes located in the mid-IR [5]. The analysis of thin polymer films in the mid-IR requires thickness ranging from 1 to 100 μm. NIR that allows the use of longer path lengths and does not require sample preparation has proved to be one of the most efficient and advanced technique for real time monitoring of polymerization reactions and quality control of manufacturing processes. Its success is attributed to the instrumental developments combined to chemometric data evaluation techniques. Thick samples can also be analyzed by attenuated total reflectance (ATR) where infrared light, introduced into a crystal (diamond being the preferred choice for its robustness) undergoes total internal reflection resulting in an evanescent wave that extends into the sample held in good contact with the crystal, at a depth of penetration typically between 0.5 to 2.0 microns. ATR has become one of the powerful and faster tools for the analysis of polymers. This approach can be coupled with macro- and micro-imaging capabilities for the characterization of compositionally complex samples such as biomedical, geological, or polymeric systems [6,7,8]. NIR and ATR are both bulk sampling techniques, but nano-IR or AFM-IR that combine atomic force microscopy (AFM) based on sharp metallized tip with standard infrared spectroscopy (IR) have launched chemical characterization of polymers to the nanometer scale [9,10]. AFM-IR provides unprecedented resolution enhancement overcoming the low spatial resolution (10–30 μm) of conventional infrared spectroscopy imposed by diffraction. This technique has paved the way to a deeper understanding and advanced chemical analysis of polymeric systems including polymer blends, polymer composites, or multilayer structures [11,12]. 

Raman spectroscopy is increasingly used for the characterization of a wide range of polymeric materials in different physical states including powders, pellets, films, fibers, liquids, and even in aqueous solutions. Combining a confocal microscope to the Raman spectrometer makes it possible to investigate the microscopic samples at different in-depth layers with a resolution in the order of 1–2 μm. Remote in situ Raman analysis by means of optical fibers is often used in industrial settings when monitoring chemical reaction processes. Regarding the identification of chemical functional groups, Raman spectroscopy complements Fourier-transform infrared spectroscopy (FTIR). For example, bands associated with double or triple bonds are much stronger in Raman spectroscopy and –S–S– and –C–S– which are important chemical groups in rubber compounds, are more readily analyzed in Raman spectra. It should be noted that the Raman effect is weak compared to Rayleigh scattering (elastic light scattered at the same frequency as that of the incident beam). The Raman scattering process can be significantly enhanced by resonance Raman spectroscopy, surface-enhanced Raman spectroscopy (SERS), or tip-enhanced Raman spectroscopy (TERS) [13]. Resonance effects occur when the energy of the photons from the exciting light matches or is close to that needed for an electronic transition of the molecule under investigation. SERS is a molecular spectroscopic technique that greatly enhances the Raman scattering of molecules adsorbed on a roughened noble-metal surface explained by the generation of a strong electromagnetic field combined with a charge-transfer state [14,15]. The TERS technique uses a sharp metal tip placed above the sample and illuminated by a laser beam resulting in strong confinement and enhancement of the electromagnetic field at the tip-apex that enhances the Raman signal scattered from the molecules in the vicinity of the apex of the scanning probe [16,17].

Details on the implementation of the two complementary vibrational spectroscopic techniques applied to polymer analysis can be found in Reference [18]. A description of the appropriate sampling techniques, as well as discussions on qualitative and quantitative analyses of polymeric samples and on important characteristics of a polymer, are given by the authors. 

It is important to mention the new development of the hyperspectral nanoimaging technology that combines vibrational spectroscopy with imaging techniques. It is based on recording the vibrational spectrum for each pixel of a 2D sample area. Amenabar et al. [19] have demonstrated the potential of hyperspectral infrared nanoimaging for mapping chemical interaction between polymer components with a spatial resolution of about 30 nm. Mäkelä and Geladi [20] have shown that hyperspectral imaging in the NIR region can be used for reliable determination of the heterogeneity of renewable carbon materials after multivariate image regression. Dong et al. [21] reviewed recent research on hyperspectral imaging based on different techniques including Raman spectroscopy for the analysis of nanoscale materials such as metal nanoparticles, carbon nanotubes, graphene, multi-layer nanoscale materials, as well as biological components in cells. 

## 3. Monitoring of Polymer Processes

Vibrational spectroscopy is particularly well suited for monitoring polymerization or curing processes. The development of fiber-optic-based spectrometers has improved the product quality by real time control of the process parameters [22]. 

Fontoura et al. [23] successfully applied NIR spectroscopy for in-line and in situ monitoring and control of monomer conversion and polymer average molecular weight in a styrene solution polymerization system. The experimental data were extracted from the region between 1150 and 1240 nm characterized by the absorption of the second overtones of the CH and CH_2_ functional groups. Evolution of the second derivative spectra of all the components of the reaction mixture was followed, and the weight fraction of polystyrene was found to be in excellent agreement with the results obtained from off-line gravimetry. 

Mid-IR and NIR spectroscopies have been used to analyze the curing process of epoxy resins. Cholake et al. [24] discussed results obtained in the literature on the curing mechanism of diglycidyl ether of bisphenol (DGABA) epoxy resin in the presence of amine curing agents by using infrared spectroscopy. The curing reaction between DGEBA and a primary amine proceeds in the first stage to the formation of a secondary amine that further transforms into a tertiary amine leading to a three dimensional network. The amount of epoxy conversion calculated from peaks located in the NIR region is higher than that found from peaks in the mid-IR. It is attributed to well separated bands in the NIR region that allow a more precise determination of the area or the height of the investigated band. 

The usefulness of Raman spectroscopy for monitoring polymerization reactions in academic and industrial environments has been demonstrated by several authors [25,26,27,28]. One major advantage of the Raman technique over infrared absorption spectroscopy is that it allows the study of aqueous heterogeneous systems such as emulsions. The emulsion polymerization of vinylacetate was followed using FT Raman spectroscopy by Ozpozan et al. [25] who used an excitation in the near-infrared range in order to get rid of the fluorescence from reaction intermediate that may mask the Raman signal. Bauer et al. [26] have shown that the on-line monitoring of a styrene-butadiene latex emulsion polymerization with the use of multivariate models provides an accurate estimation of the dry extract or of the amount of styrene monomer far more rapidly than any other analytical technique. The industrial application of Raman spectroscopy for the control and optimization of vinyl acetate resin polymerization has been discussed by Frauendorfer and Hergth [27]. Martinez et al. [28] explore the feasibility of Raman spectroscopy for polymerization reactor monitoring by using different techniques: in-line, by an immersion probe, through a site glass window or off-line by collecting samples in vials.

Vibrational spectroscopy has been applied for a long time for the study of elastomers, a class of materials widely used in the rubber industry. Of particular interest is the vulcanization or cross-linking process that chemically bridges macromolecular chains into a three-dimensional network structure in order to improve elasticity and strength. Understanding the reaction mechanisms involved in the vulcanization process allows the opportunity to tailor the formulations to the resulting properties of the final material [29]. Raman spectroscopy has proven to be well adapted for the sulfur-based curing system of unsaturated hydrocarbon rubbers on account of the sensitivity of this technique to non-polar chemical groups such as C=C and C–S [30,31]. The cross-linking process of cis-polybutadiene rubber induced by peroxide was investigated by Liu et al. [32] by means of in situ FTIR spectroscopy combined with two-dimensional correlation analysis. The authors demonstrated that the peaks at 2932 cm^−1^ and 2852 cm^−1^ attributed, respectively, to the asymmetrical and symmetrical stretching modes of the –CH_2_– groups gradually increase upon increasing the temperature from 50 °C to 220 °C, while the peaks at 3006 cm^−1^ associated with the =C–H stretching of the 1,4-structure decreased. It was concluded that a large part of the double bonds is transformed to –CH_2_– groups. At the same time, the authors observed enhancement in the intensity of the peak at 965 cm^−1^ assigned to the rocking of the trans-1,4-structure probably generated by an internal rotation.

## 4. Polymer Orientation 

Molecular orientation resulting from mechanical deformation during polymer processing can affect the physical properties of the polymeric systems. Therefore, characterizing this orientation is of particular interest for a better understanding of the mechanisms involved in polymer deformation in order to correlate the processing conditions with the properties of the fabricated sample. Several techniques, including X-ray scattering, birefringence, sonic modulus, polarized fluorescence, nuclear magnetic resonance (NMR), infrared dichroism, and polarized Raman spectroscopy have been used to quantify the level of chain orientation. Vibrational spectroscopy is particularly attractive as it provides information at a molecular level since vibrational frequencies are associated with particular chemical functional groups constituting the molecule. This allows the determination of the degree of orientation of the different components of copolymers and blends or of the crystalline and amorphous phases in semi-crystalline polymers [33]. 

Polarized Fourier transform infrared spectroscopy has been widely used for the study of polymer orientation [34,35,36]. This technique directly probes the orientation relative to a reference direction of electric dipole-transition moments associated with particular vibrational modes. It requires the determination of the dichroic behavior of a selected absorption band of the oriented sample’s infrared spectrum. Parameters used to characterize the anisotropic optical absorption of oriented species are the dichroic difference ΔA = A_//_– A**_⊥_** or the dichroic ratio R = A_//_/A**_⊥_** (A_//_and A**_⊥_** being the absorbance values when the incident infrared radiation is polarized parallel and perpendicular to the reference direction, respectively). In the case of orientational ordering occurring upon stretching uniaxially a polymer sample, the reference direction is usually taken as the direction of strain. 

**Figure 1:**
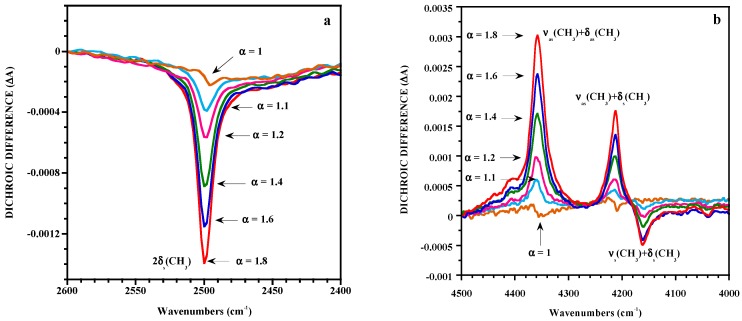
Strain dependence of the dichroic difference for a poly(dimethylsiloxane) (PDMS) film in the 2400–2600 cm^−1^ (a) and in the 4000–4500 cm^−1^ (**b**) regions. Reproduced with permission from [37].

In the classical method, measurements of A_//_and A**_⊥_**, carried out separately, are subject to instrument and sample fluctuations between the two measurements. This could affect the measurements essentially in the case of slightly oriented samples for which the dichroic difference is close to 0. The lack of sensitivity of classical linear dichroism for detecting very low dichroic effects can be significantly improved by the polarization modulation technique coupled to FTIR spectroscopy [37,38,39]. This technique uses a photoelastic modulator placed in a linear beam of light, which can alternate the polarization state of the incident radiation between directions parallel and perpendicular to the stretching axis. It has been successfully applied in the mid- and near-infrared range to investigate molecular orientation in uniaxially stretched poly(dimethylsiloxane) (PDMS) elastomeric networks that display low levels of orientation on account of the very high chain flexibility [37,39]. The dichroic behaviors of overtones and combination bands have been examined because of the strong intensity of the bands associated with the fundamental modes even for films around 100 μm thick. The reduction in intensity of overtones and combination bands allow the evaluation of dichroic effects. Figure 1 represents the dichroic difference ΔA for the band at 2500 cm^−1^ of the mid-IR and for the spectral pattern between the 4000 and 4500 cm^−1^ region of a PDMS film stretched to different draw ratios α (defined as the length of the deformed sample over its initial length). A dichroic effect can be detected for a draw ratio lower than 1.1, thus the absolute value of the dichroic difference and the orientation increase with strain. The investigated bands are associated with the methyl groups of PDMS respectively assigned to the overtone of the symmetrical bending mode and to combinations of stretching and bending modes. As seen in Figure 1b, the dichroic differences appear positively or negatively with respect to the baseline on account of the angle between the transition moment vector of the band considered and the direction of stretch. It thus appears that the dichroic behavior of the bands helps with the assignment of the observed absorptions.

**Figure 2:**
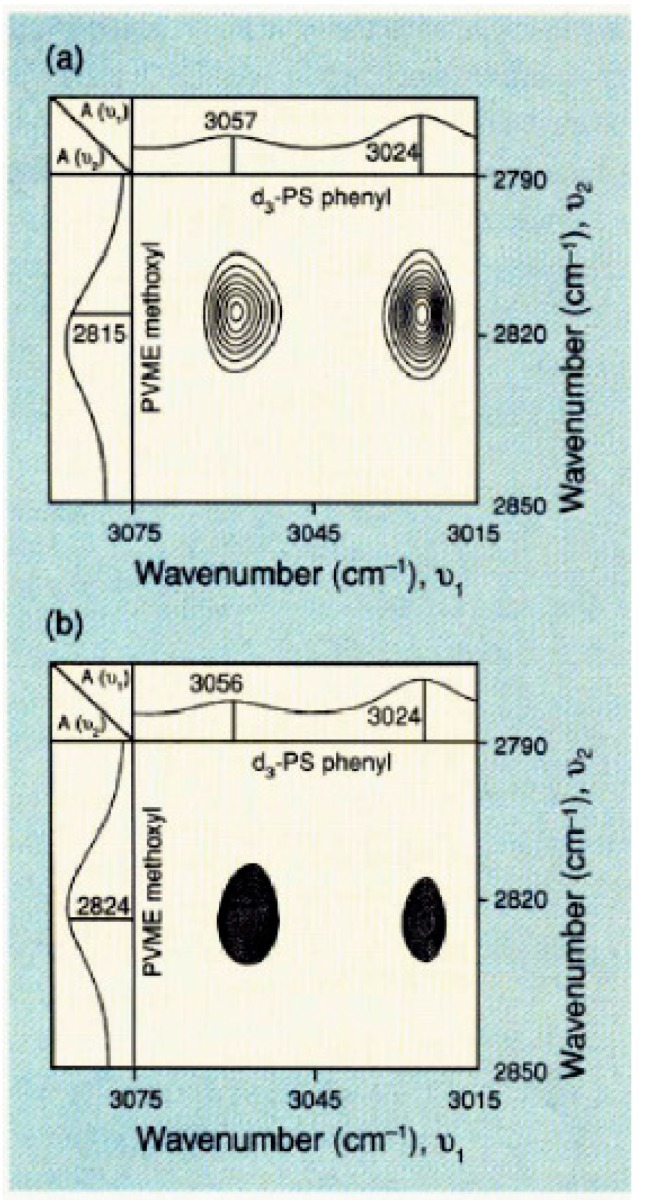
Two-dimensional infrared (2D IR) of a miscible blend (25:75) of deuterated polystyrene (d_3_-PS) and poly(vinyl methyl ether) (PVME): (**a**) synchronous; (**b**) asynchronous. Reproduced with permission from [42]. Copyright American Chemical Society (ACS) Publications, 1994.

It is of interest to mention that dynamic infrared linear dichroism in polymeric systems submitted to a small-amplitude oscillatory deformation inducing dynamic orientation response of the sample, has been used for the analysis of the time dependence of deformation, orientation, and relaxation processes. Dynamic infrared linear dichroism spectra are typically plotted in phase and in quadrature (out of phase) with the applied strain. Dynamic infrared linear dichroism coupled with 2D correlation analysis has been shown to provide insights into specific interacting sites in miscible polymer blend components. Synchronous and asynchronous spectra taken at two independent wavenumbers can be derived from cross-correlation intensities. They, respectively, represent IR signals occurring in and out of phase with each other. The reader will find more details in the papers by Noda et al. [40,41,42]. As an example, Figure 2 shows the synchronous and asynchronous 2D IR cross-correlation maps of a miscible blend (25:75) of deuterated polystyrene (d_3_-PS) and poly(vinyl methyl ether) (PVME) in the PVME methoxy CH_3_ symmetric stretching band around 2820 cm^−1^ and the PS aromatic CH stretching bands at 3024 and 3057 cm^−1^ [41,42]. Note that the backbone aliphatic hydrogen atoms of PS have been deuterated in order to avoid overlap with the PVME component. The synchronous map displays cross peaks between the PVME band at 2815 cm^−1^ and the PS aromatic CH-stretching bands at 3024 and 3057 cm^−1^, thus suggesting an interaction between the PS phenyl ring and the PVME methoxy group. The authors conclude that the electric dipole–transition moments associated with the investigated bands of the two functional groups orient parallel to each other. The asynchronous map shows a strong cross peak between the phenyl group and the band at 2824 cm^−1^, recealing a second component of the symmetric methyl stretching of the PVME methoxy group. 

Polarized Raman spectroscopy is also a powerful tool for the study of molecular orientation distributions in polymeric systems. Combined with optical microscopy, it allows the analysis of materials at the micron size scale. Furthermore, the second and fourth terms, <P_2_> and <P_4_>, respectively, of the Legendre polynomial expansion of the orientation distribution function f(θ) (where θ denotes the angle between the macroscopic reference axis usually taken as the stretching axis and the local chain axis of the polymer) can be determined, while only <P_2_> is accessible from infrared measurements. From this point of view, polarized Raman spectroscopy provides a more detailed analysis of the molecular orientation distribution. Determining the distribution of polymer chains’ orientation by polarized Raman scattering can be found in the paper by Bower [43,44], and the usefulness of this technique is demonstrated in the study of molecular orientation of various polymers [35,45,46,47]. 

## 5. Polymer Composite Characterization

The addition of filler particles to a polymer is expected to enhance the mechanical properties of the pristine medium. The state of filler dispersion, as well as the interfacial adhesion between the organic and inorganic phases are usually considered crucial parameters for the physical performance of the final materials. Reinforcing fillers are particularly needed in the rubber industry in order to increase the strength and stiffness of the elastomeric compounds which have low modulus compared to stiffer materials such as thermoplastics. While silica and essentially carbon black (CB) have been used for a long time in the tire manufacturing industry, the focus has shifted toward nanofillers of different morphology including spheres, sheets (obtained from delamination or exfoliation of layered structures), or rod-shaped particles. These nanofillers are expected to offer a larger interfacial area with the polymer, and thus a stronger reinforcing effect. The reinforcing ability of the filler particles is evaluated from the tensile stress–strain curve of the composite in comparison to that of the unfilled polymer. Studies carried out on elastomers have shown that nanofillers impart to the host matrices much higher levels of reinforcement at the same filler loading compared to those provided by the conventional carbon black or silica particles [48,49]. It has also been demonstrated that nanofillers with high aspect ratio, are by far more efficient in terms of mechanical reinforcement on account of their ability to form a filler network at a low filler content, which provides electrical conduction in the case of black fillers. It is noteworthy that the formation of a filler network is better visualized from the strain dependence of the storage modulus that exhibits a strong non-linear behavior known as the “Payne effect” [50]. Moreover, the orienting capability of these anisotropic particles in the direction of strain, strongly affects the mechanical response of the composite. However, the presence of filler aggregates acting as failure points, often reduces the strain at break. In addition to the macroscopic information provided by a mechanical analysis, vibrational spectroscopy can be used to identify the interacting species on both the macromolecular chains and the particle surface, thus allowing a characterization of fillers and filler–matrix interface. 

### 5.1. Infrared Spectroscopy

Mid- and near-infrared spectroscopies have been shown to be particularly well suited for a detailed analysis of the silanol groups (isolated and geminal) present on the surface of silica particles. These surface sites that serve as hydrogen-bonding for various chemical species, are of particular interest since they determine the surface reactivity of silica with the environment. This reactivity allows convenient chemical modifications in order to avoid hydrophilic silica particles’ aggregation and ensure adhesion with the polymer matrix. Silanol groups are considered as sites of water adsorption, and the silica–water interface can play a critical role in the reinforcement effect and in environmental processes [51]. Water molecules can be characterized by their O–H stretching band around 3700–3000 cm^−1^ and the H–O–H bending band at 1640–1630 cm^−1^. However, in the case of strong intensity of these fundamental modes, they can be identified by the combination of the bending and one of the stretching modes in the 5000–5350 cm^−1^ range. The analysis of the (ν + δ) absorption of water on silicone rubbers filled with silica particles with different surface characteristics has revealed different water–silicate interfaces [52]. FTIR has also been used to demonstrate that a simple end-group modification with a silanol functional group to one end of a styrene-butadiene chain enhances the interaction with silica particles and improves their dispersion within the host matrix [53]. Infrared spectroscopy has also been used to follow chemical modification of the surface of carbon-based nanofillers such as carbon nanotubes or graphene materials during the functionalization process intended to get a better dispersion and a stronger interfacial adhesion to the matrix [54]. 

It is of interest to mention that chemometric treatments have been successfully applied to mid- and near-infrared data for optimizing the compounding process and for a quantitative determination of composite properties. NIR spectroscopy has been used as a tool to monitor the preparation of a composite based on polypropylene and an organomodified montmorillonite, which is a layered silicate composed of stacked layers [55]. In a second paper by the same group, the interest of NIR spectroscoy for the characterization of the dispersion in polymer nanocomposites is discussed with a special focus on the application of inline techniques to monitor polymer-clay nanocomposite processing [56]. The exfoliation of the nm-thick layers and their homogeneous dispersion in a polymer are expected to provide a significant reinforcement effect of the host matrix. The use of optimized chemometric models allowed correlation of the composite’s mechanical properties (tensile strength and Young’s modulus), X-ray diffraction measurements (D-spacing), transmission electron microscopy (interparticle distance), and rheological measurements (G’ and G”) with NIR spectral data [57]. 

### 5.2. Raman Spectroscopy

Raman spectroscopy has become one of the most important tools for the characterization of carbon-based materials because they display strong resonance-enhanced Raman scattering effects making them easily identified even if dispersed in a polymer matrix at very small loadings [58,59,60,61,62,63,64,65,66,67]. 

The current study only focuses on graphene, a single layer of carbon atoms linked together in a hexagonal structure, considered as the basic unit of all graphitic carbon allotropes. Its impressive mechanical, electrical, and thermal properties have attracted an unprecedented interest as a outstanding reinforcing filler for polymeric materials. Different preparation strategies have been used in the literature to create monolayers or at least multilayer graphene formed by several graphene sheets stacked together (MLG). 

**Figure 3:**
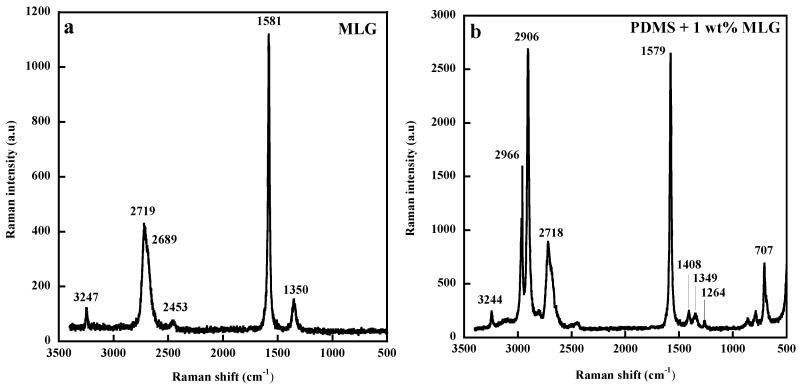
Raman spectra of multilayer graphene (MLG) (**a**) and of poly(dimethylsiloxane (PDMS) filled with 1 wt% of MLG (**b**) under excitation at 532 nm. Reproduced with permission from [63].

The Raman spectrum of MLG exhibits, in addition to the G and 2D bands at 1581 and 2719 cm^−1^ (under a 532 nm laser) present in the spectrum of a single-layer graphene, a band at 1350 cm^−1^ (D band) associated with the presence of defects or disorder (Figure 3a) [68]. The important feature in the Raman spectrum of graphene and of all carbon-based materials is the excitation-energy dependence called dispersive behavior of the D and 2D bands that up-shift with increasing photon energy. This peculiar phenomenon widely discussed in the literature [68,69,70,71] has been explained by a double-resonant Raman process that involves coupling of electrons and phonons away from the center of the Brillouin zone. The overtone of the D band, the 2D band, also present in defect-free graphite-like materials, has been shown to yield information on the number of layers and on their interactions [68]. 

Figure 3b displays the Raman spectrum of PDMS filled with 1 wt% of MLG with the D, G, and 2D bands, respectively, located at 1349, 1579, and 2718 cm^−1^. The main challenge in composites is to transfer the outstanding mechanical properties of graphene to the matrix. As discussed recently by Kinloch et al. [72], the full potential of graphene has not yet been realized, which stems from issues including poor load transfer, inhomogeneous dispersion, or high viscosity causing composite processing problems. 

**Figure 4:**
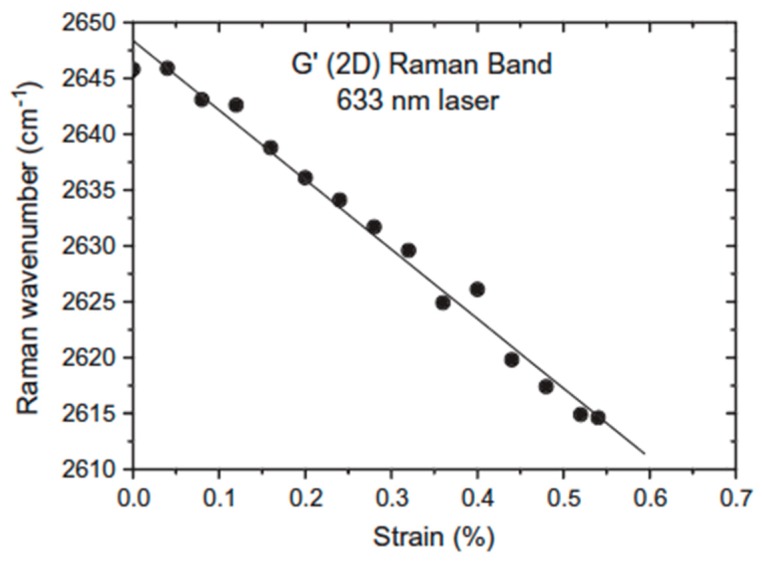
Shift of the Raman 2D band with strain for a graphene monolayer. Reproduced with permission from [75].

Raman spectroscopy has been used to follow the deformation of carbon fibers because the frequencies of the D and 2D bands of carbon fibers have been found to be sensitive to the level of applied strain [73,74]. They shift to lower wavenumbers with increasing uniaxial strain with a rate of frequency shift of the 2D band twice that of the D band. This makes the overtone of the D band quite useful for the evaluation of strain distribution in the carbon species and in their composites. Moreover, the magnitude of shift increases with the fiber modulus yielding to Raman spectroscopy the ability to probe the mechanical properties of the carbon materials. A graphene monolayer subjected to deformation shows a 2D band whose wavenumber decreases linearly with strain with a slope in the order of –60 cm^−1^/% strain that corresponds to a Young′s modulus of 1200 GPa [75] (Figure 4). However, the rate of band shift per unit strain has been shown to decrease as the number of graphene layers increases [76]. In a study devoted to the reinforcement of natural rubber by graphene nanoplatelets (GNPs), Li et al. [77] showed no large difference in the overall stress–strain curves between the GNPs and CB fillers. However, the initial slope is significantly higher in GNP-filled materials (the modulus at 100% strain is more than twice that of the CB loaded sample at a same filler content), which, in our sense, is the result of the formation of a filler network on account of the high anisotropy of graphene sheets [48]. With regard to pristine graphene, the shift rate of the 2D Raman band of GNPs in nanocomposites has been found to be very small (~−1 cm^−1^ for 100% strain) leading the authors to conclude that the stress transfer that takes place at the polymer–filler interface is relatively inefficient. We reached the same conclusion by comparing the dispersing behavior of the 2D band of MLG or multiwall carbon nanotubes (MWCNTs) in the pure state and embedded in a silicone matrix [63,65]. The excitation-energy dependence of the 2D band was found quite similar in both cases, indicating poor interactions between the polymer chains and filler particles, and showing that there is still a challenge for graphene derivatives to transfer their impressive mechanical properties and give rise to reinforcement of composites.

## 6. Conclusions

The importance of infrared and Raman spectroscopy for the analysis of various properties of polymer systems is discussed in this paper. Both techniques provide a “molecular fingerprint” of a sample and enable analysis of chemical composition and molecular structure. The basic principles as well as the most appropriate sampling techniques are recalled with a special emphasis on near-infrared spectroscopy that probes overtones and combination of fundamental modes. This technique has emerged, with the assistance of chemometrics, as one of the most powerful tools for quality control of polymer processes. It is also recommended for the analysis of thick polymer films in the transmission mode, and offers a useful way for determining chain orientation. Combined with the polarization modulation technique, it allows the detection of low dichroic effects with high precision. Raman spectroscopy has become, besides its increasing use for process monitoring and control by means of fiber optic sampling probes, one of the most important techniques for the analysis of carbon nanostructures on account of their strong resonance-enhanced scattering effects. It can be applied to all carbon-based nanocomposites for an evaluation of polymer–filler interfaces. It is noteworthy that coupling of each type of vibrational spectroscopy with AFM or scanning probe microscopy in order to increase the spatial resolution has a great potential for the characterization of polymeric structures and quantitative evaluation of some of their physical properties. Furthermore, the hyperspectral imaging technology, which has great promise for the study of nanoscale materials, will probably be nicely combined with the two vibrational techniques.
